# The role of p130Cas/BCAR1 adaptor protein in the pathogenesis of cardiovascular diseases: A literature review

**DOI:** 10.1016/j.ahjo.2024.100416

**Published:** 2024-06-25

**Authors:** Ghazal Ghasempour Dabaghi, Mehrdad Rabiee Rad, Reza Amani-Beni, Bahar Darouei

**Affiliations:** a Isfahan Cardiovascular Research Center, Cardiovascular Research Institute, Isfahan University of Medical Sciences, Isfahan, Iran; b School of Medicine, Isfahan University of Medical Science, Isfahan, Iran

**Keywords:** p130Cas, BCAR1, Breast cancer anti-estrogen resistance, Adaptor protein, Cardiovascular disease

## Abstract

Breast cancer anti-estrogen resistance-1 (p130Cas/BCAR1) is an adaptor protein of the cas(Cas) family. This protein regulates multiple complex pathways in different organs including bones, pancreas, and immune and cardiovascular systems. Although previous research well demonstrated the role of p130Cas/BCAR1 in different diseases especially cancers, a precise review study on the various effects of p130Cas/BCAR1 on cardiovascular diseases is missing. In this study, we reviewed mechanisms of action for p130Cas/BCAR1 impact, on cardiac embryonic development defects, hypertrophy and remodeling, pulmonary artery hypertension (PAH), and atherosclerosis. Also, we suggest feature direction for research and potential therapeutic implications. This study showed that p130Cas/BCAR1 can affect cardiovascular diseases in various mechanisms including actin stress fiber formation, attachment to focal adhesion kinase (FAK) and angiotensin II (Ang II), generation of reactive oxygen species (ROS), and growth factor signaling through amplifying receptor tyrosine kinase (RTKs).

## 1 Introduction

p130Cas/BCAR1 (Breast Cancer Anti-Estrogen Resistance 1) is an adaptor protein of the Crk-associated substrate (Cas) family. This protein was first discovered in 1991 [1]. Four adaptor proteins comprise the Cas family, including the p130Cas/BCAR1, Cas-L/HEF/NEDD9, EFS/SIN, and HEPL/CASSS4 [2]. These Cas family members have similar structures with several interaction domains with various molecules, mainly for protein-protein interactions and serine and tyrosine phosphorylation sites [2,3]. Multiple studies showed that p130Cas/BCAR1 regulates multiprotein signaling pathways and cell transformation that control cell migration, adhesion, cell motility, transformation, microbial pathogenesis, survival, and tumor progression. This is mainly due to its interaction abilities with focal adhesion kinase (FAK) and the Src family kinases, which are necessary for forming and disassembling focal adhesions during cell movement. Considering the role of p130Cas/BCAR1 at the cellular level this protein can be related to bone metabolism and diseases (osteoporosis, rheumatoid arthritis, bone invasion in the setting of cancer metastasis), cancer, immune system, and cardiovascular diseases (CVDs). Studies regarding the role of p130Cas have been conducted on CVDs, including cardiac hypertrophy, pulmonary arterial hypertension (PAH), and coronary artery disease (CAD) [[4], [5], [6]]. Although previous studies investigated the role of p130Cas/BCAR1 in different biologic systems, a specific comprehensive review on the cardiovascular system from the embryogenic stage to the presence of cardiovascular diseases is missing [2,7]. In this review we aim to investigate the different aspects of p130Cas/BCAR1 in cardiovascular diseases from the embryogenic development level to other CVDs, including cardiac hypertrophy, myocardial remodeling, PAH, and atherosclerosis (Table 1).

**Table 1:** The effect zones and the potential mechanisms of P130Cas.

Area of effect	Possible mechanism of action
Cardiac embryonic developments	Disrupted myofibrillogenesis, ill-formed *Z*-disks, and reduction in the formation of actin stress fibers.
Cardiac hypertrophy	Transmits mechanical and biochemical signals within cardiac cells to coordinate their response to stress, interacts with proteins at the Z-lines of sarcomeres, changes in gene expression by modulating the activity of transcription factors, and direct interaction with FAK.
Myocardial remodeling	ERK1/2 pathway, NF-κB pathway, interaction with Src family kinases, interaction with Ang II, and ROS formation.
Pulmonary arterial hypertension	Amplifying EGF-R, amplifying RTKs, amplifying PDGF-R, amplifying FGF-R, and enhanced ERK1/2.
Atherosclerosis	the BCAR1-CFDP1-TMEM170A loci on chromosome 16 effect stability of arterial wall's structural, connection with RAS and Ang II, and VSMC contractility regulation through facilitation of actin cytoskeleton.

FAK: focal adhesion kinase; ERK: extracellular signal-regulated kinases; NF-κB: nuclear factor-kappa B; Ang II: angiotensin II; ROS: reactive oxygen species; EGF-R: endothelial growth factor receptor; PDGF-R: platelet-derived growth factor receptor; FGF-R: fibroblast growth factor receptor; RTKs: receptor tyrosine kinase; VSMC: vascular smooth muscle cells.

## 2 p130Cas protein structure

**Fig. 1:**
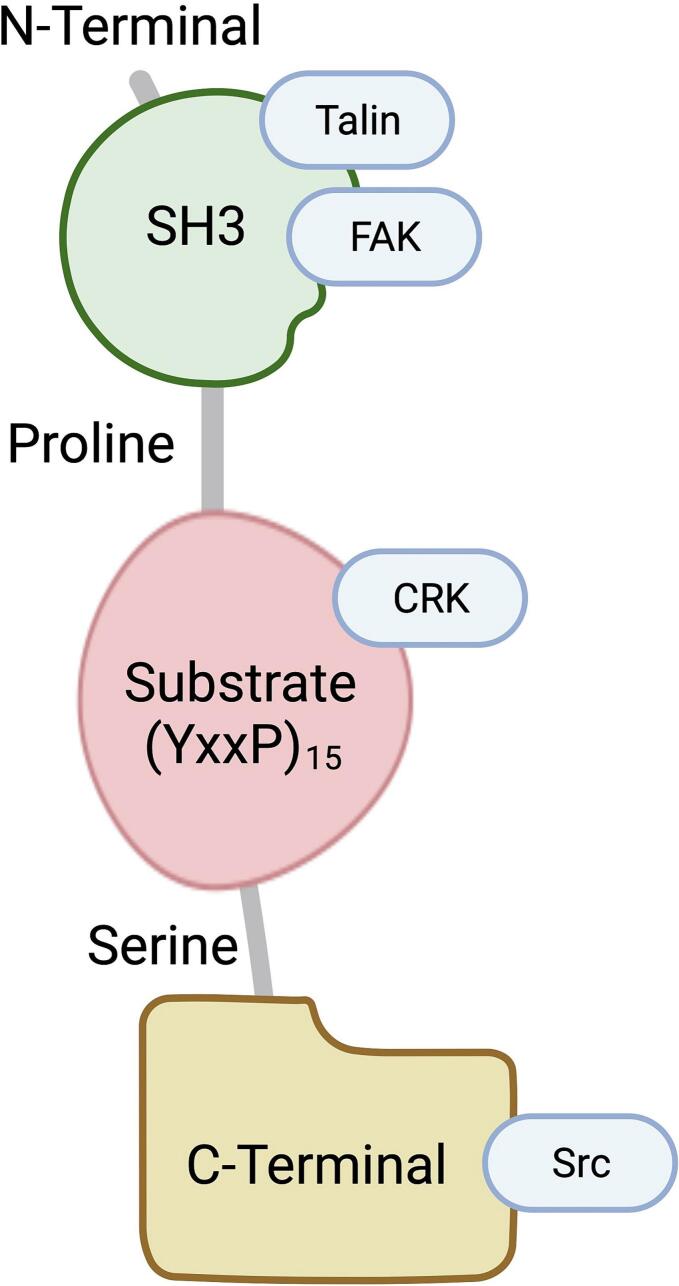
Schematic representation of p130Cas structure (Created with Biorender.com). FAK, focal adhesion kinase.

P130Cas protein can be identified by an amino (N)-terminal Src-homology 3 (SH3) domain (Fig. 1). Additionally, it possesses an adjacent large domain that is accountable for the attachment of substrate, which in this domain, there is a total of 15 repetitions of the YxxP motif. This domain is the primary location for tyrosine phosphorylation on the p130Cas/BCAR1 molecule, creating SH2-binding locations. Furthermore, a region rich in proline and serine can be observed, besides the greatly preserved four-helix bundle recognized as the focal adhesion targeting (FAT) domain [[8], [9], [10]]. P130Cas features multiple domains that facilitate protein-protein interactions, critical for its function as a signaling hub. The modulation of these interactions is often mediated by phosphorylation events on tyrosine and serine/threonine residues within the protein [10]. These post-translational modifications create docking sites for various signaling proteins, propagating cellular responses to external stimuli [11]. The phosphorylation status of p130Cas is intricately controlled by various factors such as hormones, growth factors, and mechanical forces [11]. This regulation is key to ensuring that p130Cas can fulfill its role in transmitting signals appropriately, thereby influencing the downstream processes in which it is involved.

## 3 Cardiovascular disease associated with p130Cas

### 3.1 Cardiac embryonic developments

Within the scope of cardiac embryonic development, p130Cas is essential for adequately forming the heart and embryogenesis from the early stages of cardiac myocyte differentiation to the development of complex heart structures [9,12,13]. Deficiencies in p130Cas can lead to profound developmental defects, underlining its significance in embryonic cardiovascular formation. Studies involving p130Cas-deficient mice have highlighted the importance of this protein in cardiovascular development. These mice exhibit significant impairments in formation of heart, including systemic congestion, thin myocardium, dilated blood vessels, and growth retardation, leading to lethality around 11.5–12.5 days post-coitum [12]. The defects observed in the Cas-deficient mice were not merely subtle deviations but rather profound disruptions indicative of the pivotal role of p130Cas in early heart development. The early death of p130Cas-null mice during the embryonic stage hinders the understanding of its function in both development and adulthood [9].

Honda et al. observed disrupted myofibrillogenesis and ill-formed *Z*-disks in the hearts of p130Cas-deficient mice embryos [12]. Myofibrils, the fundamental contractile units of cardiac muscle cells, are essential for the heart's functionality, and Z-disks serve as architectural beacons within myofibrils, marking the boundaries of sarcomeres and also enabling the ordered assembly of contractile proteins [14,15]. The anomalies in these structures highlight the possible roles of p130Cas in the biomechanical and signaling aspects of cardiac muscle cell formation. One particularly critical function of p130Cas in cardiac cells is the formation of actin stress fibers. These structures are important for maintaining cell shape, enabling cellular contraction, and facilitating intracellular signal transduction [16]. In p130Cas-deficient cells, there is a marked reduction in the formation of actin stress fibers, which likely contributes to the observed developmental cardiac defects [12]. The precise mechanisms by which p130Cas orchestrates the assembly of actin stress fibers and the implications of this on heart formation continue to be areas of active investigation.

**Fig. 2:**
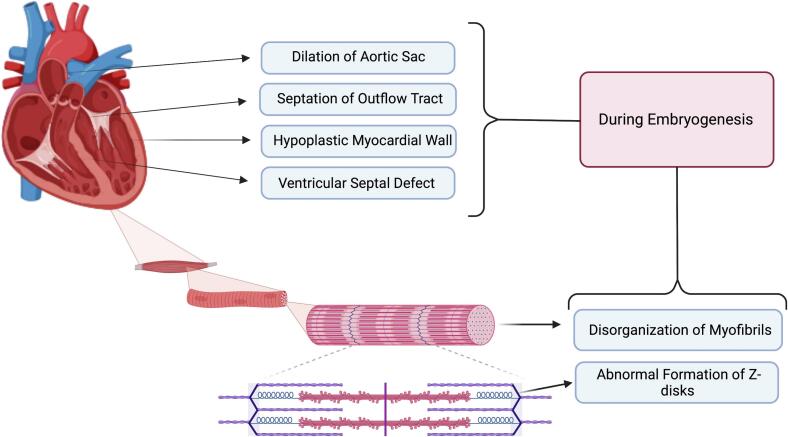
The role of p130Cas in cardiac development during embryogenesis (Created with Biorender.com).

These reports suggested that for normal embryonic development, p130Cas/BCAR1 is a key factor, however, whether defects observed in BCAR1-null embryos are caused directly by the role of p130Cas/BCAR1 in cardiovascular development or indirectly by the effect of BCAR1 on complete organismal loss is insufficient. In a recent study in 2022 by Mahmoud et al., they demonstrated that targeted deletion of p130Cas/BCAR1 using SM22-Cre mice in early smooth muscle or cardiac cells, or specifically in neural crest cells, leads to severe abnormalities, including the hypoplastic myocardial wall, impaired ventricular development, septation of the outflow tract and ventricle, leading to persistent truncus arteriosus, and abnormal dilatation and the aortic sac remodeling in cardiovascular development (Fig. 2) [13].

### 3.2 Cardiac hypertrophy

Cardiac myocyte hypertrophy represents an adaptive mechanism of the heart in response to various intrinsic and extrinsic stress-inducing factors [17]. The prolongation of cardiac myocyte hypertrophy can become harmful, causing cardiomyopathy, heart failure (HF), sudden cardiac death (SCD), and cardiac morbidity and mortality [18,19]. At the cellular level, the development of hypertrophy is reported to occur due to a combination of mechanical factors, including strain, and neurohormonal stimuli, like endothelin and angiotensin II (Ang II) [20]. One of the key molecules involved in this process is p130Cas, which transmits mechanical and biochemical signals within cardiac cells to coordinate their response to stress [5]. The p130Cas molecular mechanisms of action that contribute to cardiac hypertrophy are multifaceted and involve multiple signaling pathways.

Through interaction with various proteins, p130Cas forms part of a signaling complex that can influence the organization of the cytoskeleton and gene expression. Studies have indicated that p130Cas interacts with proteins at the *Z*-lines of sarcomeres, which are the functional units of muscle contraction [5]. These interactions appear critical for reinforcing the structural integrity of the sarcomere during hypertrophic growth [21]. The sarcomere is critical for the mechanical contraction of the heart, and FAK and p130Cas have significant roles within this architecture.

FAK and p130Cas are involved in coupling mechanical signals to biochemical ones, a process vital for cellular adaptation to mechanical stress [5]. Endothelin (a potent vasoconstrictor and hypertrophic agent) stimulation increases the tyrosine phosphorylation of CAS and FAK. This post-translational modification alters the function of these proteins and their interactions, which can lead to changes in the organization and function of the sarcomere [5]. Also, FAK is known to be crucial in mechanotransduction. It is related to converting mechanical stimuli into chemical activity [20]. Additionally, the localization of p130Cas and FAK to sarcomeric *Z*-lines indicates that these focal adhesion proteins could participate directly in organization of sarcomere and the hypertrophic process.

Moreover, p130Cas affects gene expression by modulating the activity of transcription factors. For instance, overexpression of the regions of FAK that interact with p130Cas has been shown to suppress the endothelin-induced gene expression, such as atrial natriuretic peptide (ANP) and brain natriuretic peptide (BNP), both of which are markers of cardiac hypertrophy [5]. This suggests that p130Cas can indirectly influence gene expression by its association with FAK, thus impacting the hypertrophic process. Also, FAK is known to interact with, which is crucial in mechanotransduction, the process by which cells convert mechanical stimuli into chemical activity [22].

### 3.3 Myocardial remodeling

The renin-angiotensin system (RAS) regulates cardiovascular physiology, including controlling blood pressure, fluid and electrolyte balance, and vascular tone [23]. Central to this system is Ang II, a potent vasoactive peptide that exerts various effects on the cardiovascular system [24]. Ang II signaling pathways have been implicated in myocardial remodeling, hypertension, atherosclerosis, and HF [25]. Sustained activation of the Ang II signaling pathway can contribute to pathological changes in the heart's structure and function, including cellular hypertrophy, fibrosis, and inflammation, leading to the deterioration of cardiac performance [26]. Among the various signaling molecules involved in the Ang II signaling pathways, p130Cas has emerged as an essential player in myocardial remodeling.

During pathological stress, p130Cas is upregulated and interacts with various signaling proteins, activating downstream pathways that promote hypertrophic growth of cardiomyocytes [27]. These pathways include the extracellular signal-regulated kinases (ERK1/2) and the promotion of the nuclear factor-kappa B (NF-κB) signaling cascade, which is crucial to the development of cardiac hypertrophy [28,29]. The mentioned signaling pathways, leading to hypertrophic and fibrotic responses in the heart are facilitated by Ang II. Ang II stimulates p130Cas phosphorylation and accelerates its connection with other signaling molecules like Src family kinases [27]. Also, Disruption of p130Cas function or expression can attenuate Ang II-induced hypertrophic effects, suggesting that p130Cas is a key modulator of Ang II-mediated myocardial remodeling [27].

Reactive oxygen species (ROS) are small reactive molecules with a dual role in the heart: first, they are essential for several physiological signaling processes, and second, they contribute to oxidative stress and cardiac damage [30]. p130Cas has been implicated in promoting the generation of ROS, a process that can exacerbate vascular inflammation, endothelial dysfunction, and fibrosis. The role of p130Cas in ROS generation is particularly evident in the context of Ang II signaling [27]. Ang II stimulation can activate NAD(*P*)H oxidases through p130Cas-related pathways, producing superoxide and other ROS [31,32]. These ROS, in turn, can activate redox-sensitive signaling pathways, such as activator protein-1 (AP-1) and NF-κB, which have a role in the inflammatory response and fibrotic remodeling of the heart [33,34]. The link between p130Cas-mediated effects and CVD progression becomes apparent when considering the impact of oxidative stress on vascular health. Increased ROS levels can activate matrix metalloproteinases (MMPs), degrading extracellular matrix components and contributing to cardiac tissue remodeling [27]. This process is a hallmark of HF and is associated with adverse clinical outcomes, which indicates the need for more research in this field.

### 3.4 Pulmonary arterial hypertension

PAH is a multifaceted and fatal disease distinguished by a persistent elevation in the pulmonary artery pressure (PAP) due to vascular remodeling, which can lead to right HF, reduced oxygen delivery to the body, and even death [35]. Despite significant advances in understanding the pathological mechanisms underlying PAH, there are still considerable gaps in the knowledge about its molecular etiology, which can limit the effectiveness of treatments.

p130Cas/BCAR1 is noteworthy in the context of PAH due to its over-expression in both serum and walls of distal pulmonary arteries. In this regard, Tu et al. showed the marked elevation of p130Cas in the serum of these patients compared to control subjects, suggesting a correlation between p130Cas overexpression and PAH pathology [6]. They also reported overexpression of p130Cas/BCAR1 and tyrosine phosphorylation in smooth muscle cells and endothelial muscle cells of patients afflicted with idiopathic PAH. The over-activation of p130Cas signaling has been observed in both experimental and human studies of PAH, indicating its vital role in the disease's progression. This over-activation contributes to the pathogenesis of PAH by amplifying growth factor signaling pathways, such as those initiated by endothelial growth factor receptor (EGF-R), platelet-derived growth factor receptor (PDGF-R), and fibroblast growth factor receptor (FGF-R) [[36], [37], [38]].

p130Cas is essential for transmitting signals induced by growth factors through amplifying receptor tyrosine kinases (RTKs) downstream signals. The attenuation of p130Cas/BCAR1 tyrosine phosphorylation due to RTK inhibitors can partially resolve PAH in animal models. The rat model with induced PAH by monocrotaline also yielded similar results, indicating that increased p130Cas phosphorylation enhanced subsequent signaling pathways, like ERK1/2, that lead to pathological migration and proliferation [6].

### 3.5 Atherosclerosis

CAD, a major cause of mortality worldwide, is a complex disease affected by various genetic and environmental aspects [39]. Recent advances in genetic research have highlighted the importance of genetic loci as a determinant in the pathogenesis of CAD [39,40]. One such promising locus is the BCAR1-CFDP1-TMEM170A on chromosome 16. That has been associated with subclinical atherosclerosis as a genetic determinant of the carotid intima-media thickness (cIMT) and CAD risk in individuals of European ancestry [4]. Subclinical atherosclerosis, characterized by cIMT, often precedes the clinical onset of CAD [41]. The lead SNP rs4888378 on chromosome 16 has been associated with the differential expression of genes in vascular tissues, particularly influencing the expression of TMEM170A, BCAR1, and lactate dehydrogenase D (LDHD) [4]. These genes are thought to be implicated in the start and progression of atherosclerotic changes by impacting the stability of arterial wall's structural and cellular functions [4].

Furthermore, a central element for the cardiovascular system's function is the RAS. Beyond the primary role of RAS in vasoconstriction, sodium retention, and blood pressure regulation, RAS has been implicated in the pathogenesis of various CVDs such as myocardial infarction, and atherosclerosis [42,43]. We mentioned that p130Cas is a key modulator of Ang II. This component can activate ROS, inflammatory process, platelet activation and vasoactivity, which all lead to the development of diseases such as hypertension, atherosclerosis, restenosis, and HF [27], and affects the activation of ROS in the Ang II signaling pathway earlier. The link between p130Cas,and Ang II signaling pathways is particularly relevant in atherosclerosis [27,42]. Ang II enhances the expression and phosphorylation of p130Cas, influencing smooth muscle cell function, a key component in atherogenesis [27].

The relationship between p130Cas and atherosclerosis is further supported by evidence linking p130Cas to endothelial dysfunction, smooth muscle cell migration, and proliferation, all of which are hallmarks of the atherosclerotic process [27].

p130Cas/BCAR1 has also been reported to play an important role in regulating the contractility of vascular smooth muscle cells (VSMC) through its facilitation of actin cytoskeleton reformation [43]. Studies also suggest that ROS can activate transcription factors, including NF-κB, AP-1, and nuclear factor erythroid 2–related factor 2 (Nrf2), which are involved in atherosclerosis pathogenesis [33,34]. As mentioned before, p130Cas may promote the formation of ROS, which can lead to vascular inflammation and endothelial dysfunction.

## 4 Therapeutic implications and future directions

p130Cas presents several potential targets for therapeutic intervention in CVD due to their involvement in pathophysiological signaling pathways [[4], [5], [6],^27^]. However, the challenge lies in the pleiotropic nature of p130Cas signaling; given that it is involved in various cellular processes, therapeutic strategies must be precisely targeted to avoid widespread effects on cellular function.

In the treatment field of cardiac hypertrophy, current therapeutic approaches often focus on managing risk factors (e.g., hypertension) or blocking the action of neurohormonal factors However, drugs that modulate the function or expression of p130Cas or interfere with its downstream signaling pathways could offer novel treatments for cardiac myocyte hypertrophy [5]. In the field of Ang II, a more detailed knowledge of how p130Cas contributes to myocardial remodeling and its interaction with Ang II signaling could inform the development of more targeted therapies [27]. Besides, inhibition of the Ang II type I receptor (AT1R) has been a widely utilized strategy in controlling hypertension and mitigating atherosclerosis-associated risks. Targeting p130Cas, or its downstream effectors, could provide a means to specifically attenuate the maladaptive cellular responses to Ang II without disrupting the physiological functions of the RAS [27]. Research on small molecule inhibitors, neutralizing antibodies, or genetic strategies to modulate p130Cas activity is in its early stages. However, it holds promise for treating atherosclerosis and potentially other CVDs where Ang II and p130Cas are pathological.

The understanding that p130Cas is integral to the pathological signaling in PAH has brought attention to the possibility of targeting this molecule therapeutically. Inhibiting EGF, FGF, and PDGF receptors has shown potential in treating PAH, as these pathways are often amplified by aberrant p130Cas activity [6]. Attenuating the abnormal increase in p130Cas and the activation of associated signaling pathways, such as ERK1/2, are strategies that could mitigate the adverse cellular processes driving PAH [6]. Pharmacological inhibition of p130Cas has been suggested to induce regression of established pulmonary hypertension, providing a hopeful outlook for patients with advanced stages of the disease.

Significant breakthroughs have been made in the role of p130Cas; however, more research is necessary to examine its function in developing targeted therapies thoroughly. Future research directions include identifying specific p130Cas-mediated signaling modules amenable to pharmacological manipulation to regulate p130Cas expression or function. Thus, the continued investigation into p130Cas and its signaling networks holds promise for advanced treatment options in managing CVDs. More detailed examination on the relation between P130Cas and PAH, and possible molecular mechanisms leading to PAH may lead to finding potential treatment options for this disease. As we mentioned earlier, there is specially a lack of effective treatment for PAH which may be because of the gaps in the knowledge about its molecular etiology.

## 5 Conclusion

**Fig. 3:**
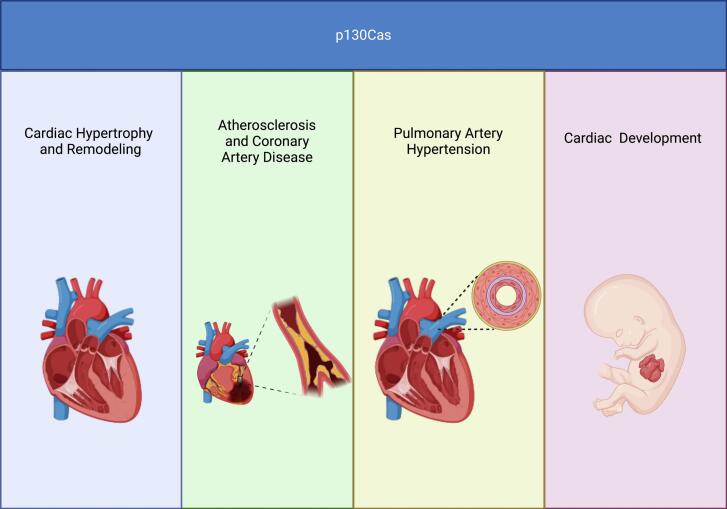
Cardiovascular diseases associated with p130Cas protein (Created with Biorender.com).

The role of p130Cas in CVDs is multifaceted and intricately linked to cardiovascular health and disease pathogenesis. The p130Cas is involved in different signaling pathways, such as cell migration, adhesion, cell motility, transformation, microbial pathogenesis, survival, and tumor progression, highlighting its significance as a nodal point for therapeutic interventions. In the field of CVDs, the potential to develop treatments that specifically inhibit p130Cas could influence the management of myocardial hypertrophy and remodeling, PAH, and atherosclerosis, offering new hope for patients suffering from these debilitating diseases (Fig. 3). Future studies should further indicate the exact role of p130Cas in CVDs to fully evaluate its potential for developing novel cardiovascular therapies.

## Ethical statement

The data of this review is based of previously conducted articles. It does not present data nor experiments with new human participants and animals performed by the authors.

## CRediT authorship contribution statement

**Ghazal Ghasempour Dabaghi:** Writing – review & editing, Writing – original draft, Conceptualization. **Mehrdad Rabiee Rad:** Writing – review & editing, Writing – original draft, Supervision, Conceptualization. **Reza Amani-Beni:** Writing – review & editing, Writing – original draft. **Bahar Darouei:** Writing – review & editing, Writing – original draft.

## Declaration of competing interest

The authors declare that they have no known competing financial interests or personal relationships that could have appeared to influence the work reported in this paper.
